# BBSF: Blockchain-Based Secure Weather Forecasting Information through Routing Protocol in Vanet

**DOI:** 10.3390/s23115259

**Published:** 2023-06-01

**Authors:** Hamza Sohail, Mahmood ul Hassan, M. A. Elmagzoub, Adel Rajab, Khairan Rajab, Adeel Ahmed, Asadullah Shaikh, Abid Ali, Harun Jamil

**Affiliations:** 1 Department of IT, University of Haripur, Haripur 22620, Pakistan; hamzasohail6413@gmail.com (H.S.); adeel@uoh.edu.pk (A.A.); 2 Department of Computer Skills, Deanship of Preparatory Year, Najran University, Najran 66446, Saudi Arabia; mahmood.mscs@gmail.com; 3 Department of Network and Communication Engineering, College of Computer Science and Information Systems, Najran University, Najran 61441, Saudi Arabia; meabdullah@nu.edu.sa; 4 Department of Computer Science, College of Computer Science and Information Systems, Najran University, Najran 61441, Saudi Arabia; adrajab@nu.edu.sa (A.R.); kdrajab@nu.edu.sa (K.R.); 5 Department of Information Systems, College of Computer Science and Information Systems, Najran University, Najran 61441, Saudi Arabia; asshaikh@nu.edu.sa; 6 Department of Computer Science, University of Engineering and Technology, Taxila 47050, Pakistan; 7 Department of Computer Science, Govt. Akhtar Nawaz Khan (Shaheed) Degree College KTS, Haripur 22620, Pakistan; 8 Department of Electronic Engineering, Jeju National University, Jejusi 63243, Republic of Korea; harunjamil@hotmail.com

**Keywords:** weather forecasting, vehicular ad hoc network, routing, security

## Abstract

A vehicular ad hoc network (VANET) is a technique that uses vehicles with the ability to sense data from the environment and use it for their safety measures. Flooding is a commonly used term used for sending network packets. VANET may cause redundancy, delay, collision, and the incorrect receipt of the messages to their destination. Weather information is one of the most important types of information used for network control and provides an enhanced version of the network simulation environments. The network traffic delay and packet losses are the main problems identified inside the network. In this research, we propose a routing protocol which can transmit the weather forecasting information on demand based on source vehicle to destination vehicles, with the minimum number of hop counts, and provide significant control over network performance parameters. We propose a BBSF-based routing approach. The proposed technique effectively enhances the routing information and provides the secure and reliable service delivery of the network performance. The results taken from the network are based on hop count, network latency, network overhead, and packet delivery ratio. The results effectively show that the proposed technique is reliable in reducing the network latency, and that the hop count is minimized when transferring the weather information.

## 1. Introduction

A wireless network connects groups of stationary or moving vehicles in a vehicular ad hoc network (VANET). Initially designed to ensure driver safety and comfort in vehicular settings, VANET is now viewed as an infrastructure for the intelligent transport system (ITS), with the increasing numbers of autonomous vehicles and the need for internet connectivity in various activities [1]. Moreover, VANETs offer the possibility for computers installed in stationary vehicles, such as those parked in airport lots, to serve as a resource for a network of mobile computers with minimal reliance on the internet infrastructure. VANETs consist of three primary components, the onboard unit (OBU), the road unit (RSU), and the reverse network, and these fall into two main categories: vehicle-to-vehicle (V2V) and vehicle-to-infrastructure (V2I). V2V communication forms a wireless network that enables cars to exchange information, including safety warnings and traffic updates, and transmit data about their location, speed, displacement, direction, and stability [2]. The exchange of critical data between vehicles and road infrastructure through wireless communication is known as V2I. This communication is unidirectional, with the road infrastructure transmitting information to all vehicles. A proposed application of this system is to provide secure weather forecast information using VANETs.

**Figure 1:**
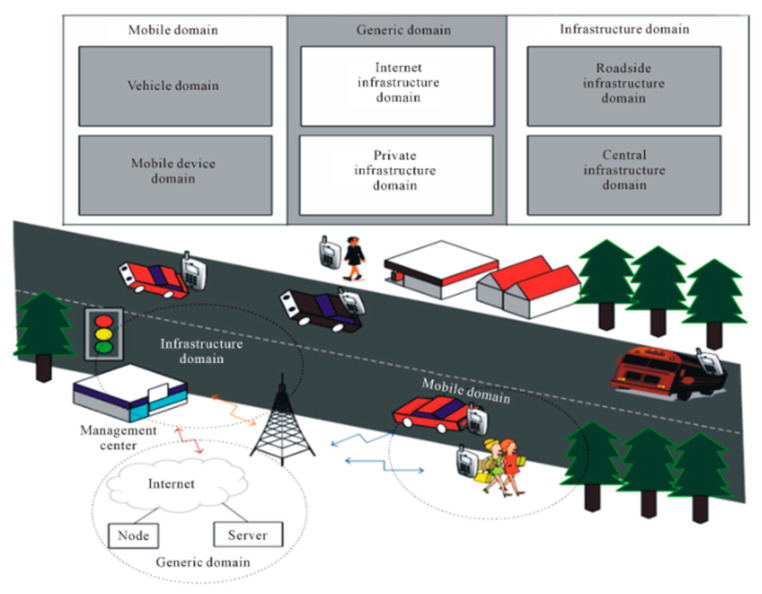
Vehicular Ad Hoc Network Communication Architecture [4].

A VANET is a type of wireless multi-hop network (WMN) that facilitates quick technological advancements in diverse infrastructure management and improves the performance of various resources even in high-mobility scenarios. A VANET employs wireless communication and computing technologies in devices for intra-vehicle and inter-vehicle communication. Inter-vehicle communication via caching is a prospective domain for standardization, research, and technological development [1]. Vehicular ad hoc networks (VANETs) offer a broad spectrum of potential applications, including collision avoidance, safety measures, navigation scheduling, real-time traffic monitoring, and more. In addition, VANETs can provide internet connectivity to vehicular nodes, which is another significant use case. Figure 1 presents an illustration of a VANET [3].

The process of weather forecasting involves using technology and scientific knowledge to observe meteorological conditions and predict atmospheric conditions in a specific location. In short, it allows for the anticipation of weather events, such as cloud cover, precipitation, wind speed, and temperature, before they occur. This forecasting is essential for people to prepare for and minimize the impact of natural disasters such as floods and typhoons, which can result in property damage and fatalities. Military operations also benefit from weather forecasting in the ability to plan around expected weather conditions to maximize chances of success [5]. In VANETs, weather forecasting is crucial in helping vehicles select routes with favorable weather conditions. For example, if there is bad weather, such as heavy rainfall or flooding, VANETs can inform nearby vehicles to decide to change their routes [6].

A set of rules known as routing protocols are utilized by routers to enable communication between the source and destination. Rather than physically moving the source’s information to its destination, routers have the sole objective of updating the routing table containing valuable data. Routing protocols serve various purposes, such as in selecting the most efficient path, preventing routing loops, enabling rapid convergence, reducing update traffic, being easy to configure, adapting to changes, scaling for large networks, being compatible with existing hosts and routers, and supporting variable lengths. In the proposed system, IPSec is employed to secure weather forecasting information in VANETs. IPSec is a suite of protocols standardized by the Internet Engineering Task Force (IETF). It operates across the IP network to authenticate, maintain data integrity, and ensure confidentiality between two communicating points. Additionally, it outlines the processes for encrypting, decrypting, and authenticating packets [7].

The main idea is to provide secure weather forecasting information among all the trustful smart vehicles. In our approach, the main content delivery is to provide security in terms of weather data. In VANETs, the best possible vehicle route is also necessary, similar to other security parameters, including security, privacy, and accuracy. Good weather is also a factor that compels a vehicle to follow an appropriate route. Weather forecasting between the V2V and V2I is needed to notify the vehicle about which suitable route would be good to travel on. We designed secure weather forecasting information flow to secure V2V and V2I communication.

Inside the VANET, the secure and reliable transmission delivery of the contents is applied. The delivery should be efficient and reliable through security parameters such as trusted nodes evaluation, nodes transmission rate, and encrypted weather forecasting information; the advantages of the mobility of the VANET support the efficient delivery of content management. We proposed an efficient, secure routing protocol to minimize the weather content request and delivery delay. The vehicles are smart enough to provide secure routing and content placement for content reliability. Reliability is one of the most important content placement and management ingredients. The routing enables us to deliver the contents to the right node with secure transmission. We have less infrastructure involvement and use distributed environments and secure content delivery.

Smart vehicles/devices are equipped with more advanced communication capabilities, such as GPS, sensors, and processing power, which enable them to collect and process weather data about the environment and other vehicles. These devices can analyze the weather data and make decisions based on the analysis. Smart devices can also communicate with other devices and the infrastructure to exchange information and coordinate actions. Examples of smart devices in VANETs include vehicles, RSUs, and sensors. On the other hand, dumb devices have limited communication capabilities and cannot process weather data or make decisions independently. These devices rely on infrastructure and smart devices to provide information and make decisions. Examples of dumb devices in VANETs include traffic lights, road signs, and cameras. Smart devices have access to various types of data and sensors that dumb devices do not have access to. For instance, in the case of weather forecasting in VANETs, smart devices can collect real-time data on temperature, humidity, air pressure, wind speed, and precipitation. These devices can also use GPS sensors to collect location data, which is critical in predicting weather conditions accurately.

In contrast, dumb devices, such as traditional cars, lack smart devices’ sensors and data collection capabilities. They cannot collect real-time data on weather conditions or provide information on the current state of the road. As such, they cannot contribute to the forecasting and dissemination of weather information.

Weather forecasting data are distributed among all the vehicles which are part of the VANET environment. The data should be secure in order to keep all information in a secure form. The nodes, which are part of the VANET environment, should be enhanced and provide secure content delivery. The main data are saved on the trustable minor nodes and shared among all the trustable and secure parts of the VANET. The main idea is to provide secure weather forecasting information among all the trusted smart vehicles. In our approach, the main content delivery is to provide the security of weather data. In VANETs, the best possible vehicle route is also necessary, similar to other security parameters, including security, privacy, and accuracy. Good weather is also a factor that compels the vehicle to follow an appropriate route. Weather forecasting between the V2V and V2I is needed to notify the vehicle of the most suitable route to travel on. We designed a secure weather forecasting information flow to secure V2V and V2I communication.

### 1.1. Motivation

Although VANET provides an efficient environment for any type of data, such as video, audio, information packets, control packets, and weather information, the management of content delivery through a VANET provides secure and efficient content management for weather information. It is necessary for smart vehicles to share weather information with other smart vehicles. Secure weather information protects smart vehicles and drivers from accidents and other incidents. V2V and V2I communication should provide secure weather forecasting and weather information handling in VANETs.

### 1.2. Importance of the Research

Weather forecasting data are distributed among all the vehicles which are part of the VANET environment. The data should be secure to keep all information in a secure form. The nodes which are part of the VANET environment should be enhanced and provide secure content delivery. The main data are saved on the trustable minor nodes and shared among all the trustable and secure parts of the VANET. The main idea is to provide secure weather forecasting information among all the trusted smart vehicles. In our approach, the main content delivery is to provide the security of weather data. In VANETs, the best possible vehicle route is also necessary, similar to other security parameters, including security, privacy, and accuracy. Good weather is also a factor that compels the vehicle to follow an appropriate route. Weather forecasting between the V2V and V2I is needed to notify the vehicle of the most suitable route for it to travel on. We designed a secure weather forecasting information flow to secure V2V and V2I communication.

### 1.3. Problem Description

During the task scheduling, in this paper, we have addressed the questions listed below:

(i). How is secure routing achieved in VANETs?
(ii). How is weather forecasting information communicated in the VANET transmission model?
(iii). How can the information requests in VANETs be managed?

### 1.4. Research Contributions

The proposed system’s main contribution are in task management and data handling with VANETs. The specific contributions are listed below:

- The security of the weather information is enhanced, and secure communication is possible.
- The system’s routing is possible through the effective delivery of weather information.
- IPsec enhances secure weather forecast information through an efficient routing scheme.

In Section 2, we outline a review of the current literature, conducted to support the proposed research methodology. We explain the current routing protocols and define routing information retrieval. Next, in Section 3, we define the proposed methodology, with the diagram and algorithm explained in the proposed methodology. In Section 4, we explain the results discussed and then, in Section 5, we explain the thesis summary. Finally, Section 5 explains the conclusions and future work cited in the proposed methodology.

## 2. Literature Review

Blockchain technology has transformed how data are stored and shared by offering a decentralized and secure transaction platform. In recent years, researchers have explored the potential of blockchains in vehicular ad hoc networks (VANETs) to improve the security and efficiency of data sharing. This proposed research focuses on utilizing blockchain technology to provide secure weather forecasting information in VANETs. The proposed system, Blockchain-Based Secure Weather Forecasting (BBSF), employs a routing protocol that enables data transmission among vehicles in a VANET. The BBSF system collects real-time weather data from multiple sources, such as weather stations and sensors mounted on vehicles, and then securely transmits the data among vehicles in the VANET [8]. The routing protocol ensures that the data are delivered to the intended recipients securely and efficiently.

The use of blockchain technology in BBSF offers several advantages. Firstly, the decentralized nature of the blockchain ensures that the data are not controlled by a single entity, thereby increasing its trustworthiness. Secondly, the tamper-proof nature of the blockchain ensures that the data cannot be altered or manipulated by malicious actors, thereby increasing its security. Finally, the use of smart contracts in blockchains enables the automatic execution of predefined actions based on certain conditions, thereby improving the efficiency of the system [9]. The proposed BBSF system has significant implications for various domains, such as transportation, logistics, and emergency response. The secure and efficient transmission of weather data in a VANET can enable better decision-making by drivers and traffic management authorities, leading to safer and more efficient transportation. Overall, the proposed research offers a novel approach to utilizing blockchain technology to improve data sharing security and efficiency in VANETs.

In VANETs, the best possible vehicle route is also necessary, similar to other security parameters, including security, privacy, and accuracy. Good weather is also a factor that compels the vehicle to follow an appropriate route. Weather forecasting between the V2V and V2I is needed to notify the vehicle of the most suitable route for it to travel on. We designed a blockchain-based secure weather forecasting information flow to secure V2V and V2I communication [10,11].

In [12], reliable and efficient alarm message routing is a real-world channel proposed for VANETs. It uses a very small number of broadcast packets to propagate alarm messages. For nodes in VANETs, it calculates the propagation of alarm messages. In REAR, two approaches are analyzed for utilizing the receipt probability. Three functions are analyzed for contention delay calculation. With the help of theory and simulations, it provides much higher reliability and contention delay using fewer broadcast packets than location-based algorithms. The authors in [13] present a secure SDN-based routing protocol for internet of vehicles uses in distributed SDN architecture, and a blockchain to secure Li and efficiently route the packets. It uses two different implementations. The first method uses SDN in the RSU network. The second method deploys SDN in the entire IOV. It uses a blockchain for securing these routing protocols. SURFER has outperformed two protocols, QRA and SD-IOV, regarding latency packet delivery and network overhead. In [14], the authors provide a VANET that enables the communication between vehicles about environmental information and alerts. This communication must be securely and correctly transmitted. A trust score evaluation scheme for secure routing in VANETs is proposed to find anonymous transport and the optimal route for the message to its destination point. The packet delivery ratio and delay are the main factors or parameters that are focused on. The message is sent in optimal five continuously, until a bogus vehicle is found. If found, the vehicle will be removed, and the next optimal path will be used. This scheme provides higher packet delivery with minimum delay.

In [15], the modern intelligent transport system has brought plenty of opportunities for VANETs. Along with this, a number of challenges also emerge. Due to this, a practical and reliable security scheme is needed to handle such challenges while protecting user privacy at the same time. 5G Technology is best for our ultra-reliable and low-latency wireless communication services and SDN architectures. These two approaches aside, they have paid for an innovative security scheme and decentralized and immutable blockchain technology. Real-time cloud-based video reports and trust management on vehicular messages provide solutions for the discussed challenges, such as malicious node detection. Video, in real time, is recorded, encrypted, and uploaded. Pictures are also used to check whether the situation is match-related or not. The results showed that the detection of malicious nodes has significantly improved. In [16], the authors introduce many protocols that have been proposed considering VANETs’ characteristics. The protocol based on vehicle position is found to be the most adequate. Urban and highway environments are studied, and different protocols are compared, such as topology and position-based protocols [17]. The comparisons are made from different perspectives regarding which protocol is used in which environment. Cycles mainly make the transition from urban to highway environment. Therefore, the transition of protocol for different scenarios might not be a good idea because the vehicle might lose its packets or connectivity in this case. Therefore, a new hybrid protocol must be introduced and developed to adapt from one environment to another [18].

The authors in [19] suggest a distributed edge-fog node-based video surveillance system for smart homes that employs motion detection and reversible blurring to protect privacy. The system interfaces with a private blockchain network to ensure integrity and offer authorization, and it is resource-efficient. In [20], the authors explore the positive and negative effects that technology, particularly in terms of a distributed ledger or blockchain, has on social animal behavior. It examines this technology’s characteristics, benefits, drawbacks, difficulties, and potential uses in various fields, including banking, health, travel, and others. In another article published [21], the author’s use of blockchain technology to guarantee data accountability and improve privacy and accessibility in Internet of Things (IoT) devices is discussed as a potential benefit. It investigates several IoT topologies and suggests a novel method that outperforms current cryptography algorithms and is secure from known vulnerabilities. The essay also thoroughly evaluates the suggested scheme’s simulation findings. This article suggests a blockchain-based alternative to improve security and transparency in the distribution of COVID-19 immunization vials, with smart contracts and proof of delivery, scalability, and throughput improvements [22]. To increase the mobile server utilization, lower the costs, and improve the performance for various applications, including augmented reality, e-transportation, e-healthcare, and education, the paper proposes a dynamic decision-based task-scheduling approach for microservice-based mobile cloud computing applications.

The suggested method successfully lowers expenses while enhancing application startup, resource use, and task arrival times [23,24]. Driving accidents can be exacerbated by diabetes. To decrease accidents, this study suggests a unique method for monitoring diabetic drivers that uses wearable sensors, machine learning, and VANET technology. The method with the highest accuracy, ranging from 90.3% to 99.5%, was random forest [25]. Another research paper [26] suggests a machine-learning-based real-time malicious node detection system for the vehicular ad hoc network (VANET), which has 99% accuracy in identifying DDoS attacks. The system was assessed using machine learning models and a distributed multi-layer classifier. The number of network nodes did not impact the system’s performance because it was set up using Amazon Web Services. To improve content distribution and management, this study suggests a machine learning-based, zonal/context-aware, equipped-content pre-caching technique for VANET. The suggested methodology improves accuracy and cache hit ratio while lowering average delay and network congestion [27,28]. Another research paper implemented in this context focused on solving issues with overheads, resource utilization, slow startup times, and the expense of running mobile applications. This study suggests a dynamic decision-based task-scheduling technique for microservice-based mobile cloud computing applications (MSCMCC). The suggested approach increases mobile server utilization while successfully lowering the cost of various applications [29].

The presented literature focused on developing different algorithms for routing, secure transactions, and communication between the vehicles and RSU. Although multiple methods and models are presented, secure weather forecasting information through routing techniques has not been presented in the literature. Redundancy, latency, collision, and incorrect message reception towards the destination are all potential effects of a VANET. One of the most crucial types of information for network control is the weather, which offers a better network simulation environment. The two main issues found within the network are packet losses and network traffic delays. Based on the discussion above, in the literature we present novel weather forecasting information through secure routing with efficient transmission in VANETs. In the next section, we present the proposed methodology.

## 3. Methodology

In this section, we define the proposed methodology at the initial stages, then we define the context of the work using the proposed three algorithms. Next, we discuss blockchain-based secure and efficient routing information through network support technology. Initially, we define the clusters to effectively enhance the workflow and support the routing protocol to travel the data securely with sufficient detail. Roadside units (RSUs), vehicular, and the blockchain-based distributed Hyperledger Sawtooth framework is used to deploy the blockchain services to secure vehicle weather forecasting information through RSU. The main functionality of the system is to adopt the routing protocol to share the secure weather forecasting network and congestion handling in the specified network parameters. We consider the V2I and V2V communication models in the proposed approach.

In our proposed system, we have secured the weather forecasting data communication between V2V and V2I in VANETs. In the first section, we extracted the data from the weather forecasting servers and monitoring systems. The OBU of vehicles has different sensory modules, including weather sensors. With the help of these sensors, the related weather data are extracted from different web servers and weather monitoring systems. In the second section, we secured the extracted data using routing protocols and, in the last section, we transmitted the secured data among trusted vehicles, roadside units, and all the nodes connected or working in the VANETs.

### 3.1. Proposed Architecture

**Figure 2:**
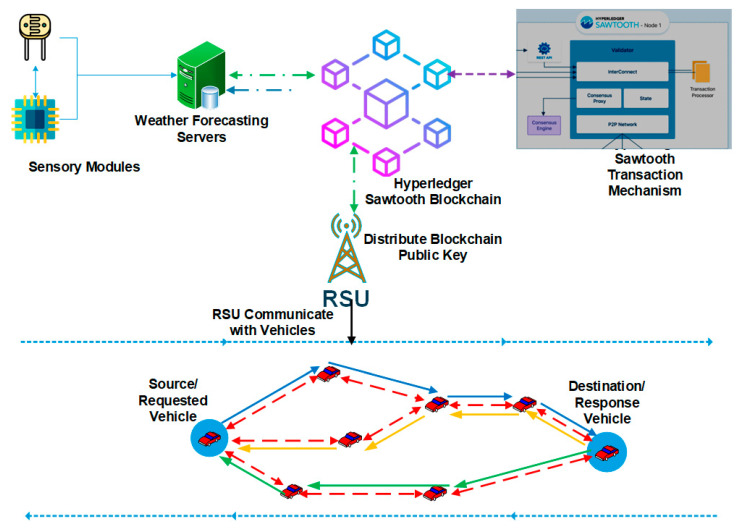
Secure routing for weather forecasting information through blockchain.

In this technique, we adopted the blockchain-based weather forecasting network modules to allow RSU to communicate directly with vehicles to follow the routing scheme with the secure public key distribution. In this approach, a source node can request the secure content. Initially, we set up all the vehicles to be trusted and to be part of the network. The trust parameters were previous messages, request rate, time spent in the network, and speed [1]. To adopt the network routing and provide the secure and reliable delivery of the content, the terms below were part of the core network. Figure 1 shows the system architecture with complete methodology. Figure 2 shows the proposed architecture with secure routing for weather forecasting information through the blockchain.

#### 3.1.1. Network Tunneling

“Encapsulation” refers to the use of a communication protocol to transfer data from a private network to a public one. This protocol enables data movement between different networks and facilitates the transmission of private network communication over a public network; although, in this approach, network tunnelling is adopted when a vehicle comes from the range of another network towards this proposed network. The network parameters adopted, that allow for communication among such parameters, are part of the central network. Based on the single request and response parameters, the proposed system allows us to communicate among these multiple vehicles.

#### 3.1.2. Weather Sensors

Devices that measure multiple parameters such as wind speed and direction, precipitation, barometric pressure, temperature, and relative humidity. The sensors capture data such as temperature, humidity, wind direction, wind speed, and rain fall. All these data are captured from weather sensors. After capturing, the data are transferred to the weather sensory modules to check the actuality of the data with the provided dataset schemes in order to enhance the dataset operations and provide the proper weather forecasting network estimation. The next part of the network is the sensory module which captures the weather data from sensors. Initially, these data are not in proper order and form, so we have to provide a reliable module to capture the data from the network.

#### 3.1.3. Weather Sensors

The common instruments of measurement are an anemometer, wind vane, pressure sensor, thermometer, hygrometer, and rain gauge. The weather measurements are formatted in a special format and transmitted to WMO to help the weather forecast model. The sensory module captures the initially investigated data from the other modules and provides reliable data in a finalized order. The sensory module describes the working with the proper order of the network estimation parameters. The proposed approach enhances the working of the provided dataset with the proposed methodology. Sensory modules relate to weather sensors to promote the proper weather forecasting network parameter settings and enhance the complete functionality of the weather data.

The proposed model used the novel implemented topology, which makes it different from other proposed models in the VANET. It is a novel research topic that we resolved in this research. The vehicle’s and the RSU-based communication architecture enhances the request and response architecture in a VANET. A detailed explanation of the proposed model is elaborated below.

**Figure 3:**
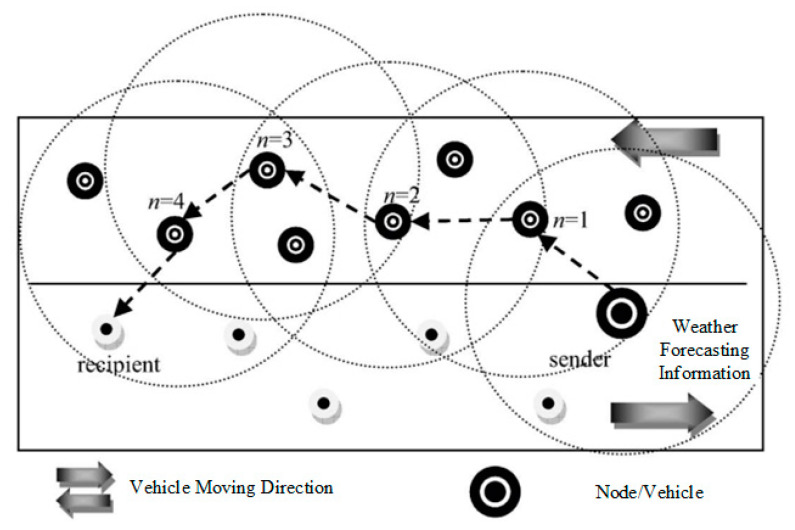
Weather forecasting information sending and receiving in the proposed methodology.

**Figure 4:**
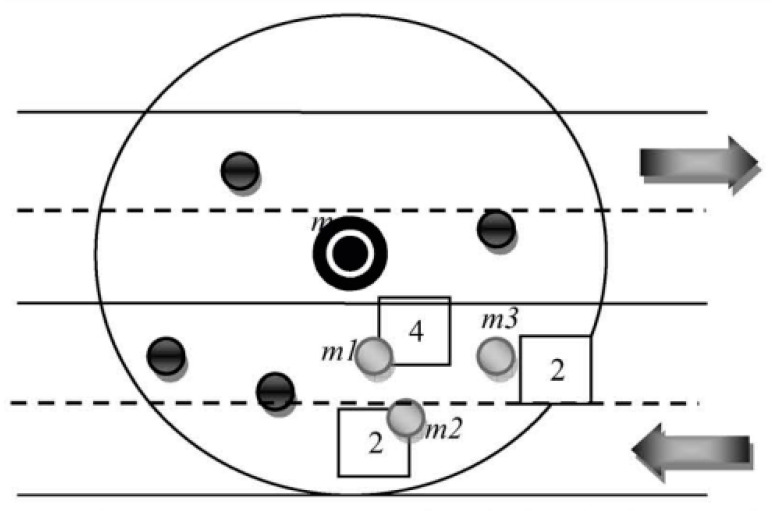
The time reservation method for efficient routing and effective node receives the reservation point when receiving the packets forwarded.

**Figure 5:**
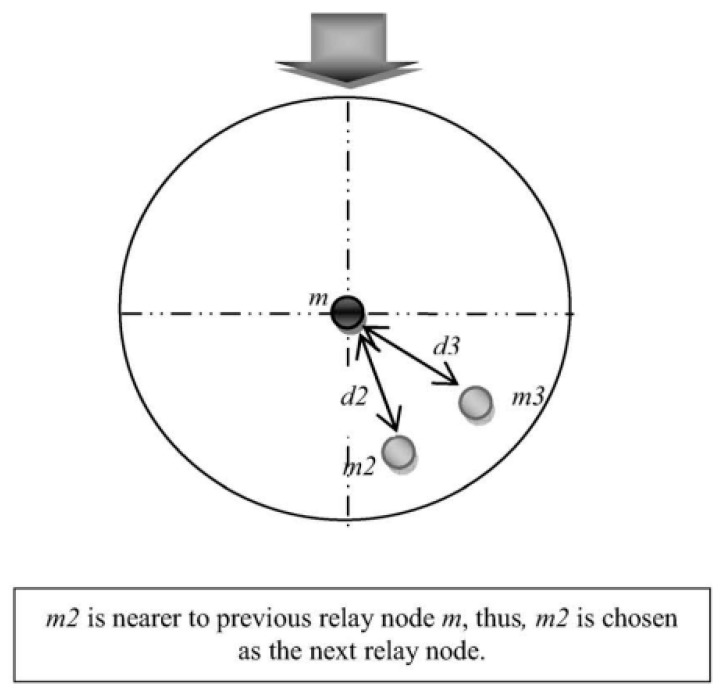
This distance-based method computes the intermediate node distance from the border of the current vehicle domain.

- Initially, we set up the base topology using a 5G network under the highway that connected the vehicles using the communication protocols.
- The weather sensors were equipped with RSUs, and vehicles travelling in both directions captured the weather data and forwarded the data to the weather forecasting server.
- The weather forecasting server is connected using the Hyperledger Sawtooth blockchain transaction mechanism to connect and control the transactions. The transactions contain secure weather forecasting information on the road the vehicles are moving towards.
- The weather forecasting information is distributed to all the vehicles connected to RSUs with a distributed blockchain public key to display weather information.
- The private key is only distributed to the vehicles which are part of the network.
- The source-to-destination information is routed based on the methodology presented in Figure 3, Figure 4 and Figure 5 and Algorithm 1.

### 3.2. Weather Forecasting Servers

Weather forecasting servers were part of the services offered through the proposed methodology. These servers were set up through highly efficient machines, and provided effective and reliable content management and processing delivery. They captured the data coming from the sensor modules and weather forecasting units. The server deployed a blockchain Hyperledger Sawtooth transaction mechanism to secure the routing and weather forecasting information. The purpose of the security in this section was to enhance the working features of the proposed technique. The working of the weather forecasting server is as follows:

- Initially, it received the weather forecasting information from the sensory modules and stored it in memory. The record for every entry is stored in a separate location using log files kept for future data searching.
- The server periodically checks the data coming from sensory modules for weather forecasting information. The network setting and road conditions are also checked based on the provided setting and reliable content delivery framework.
- After gathering the data from sensory modules, the blockchain-based Hyperledger Sawtooth framework is applied to secure the provided data. The blockchain framework provides two types of communications: one is in the form of secure and trusted routing, and the second is in the form of secure weather information.
- In this section of the thesis, we take the routing and pre-secured framework and enhance the working of the proposed approach through secure routing and weather forecasting frameworks.
- The public keys are distributed among networks for decrypting the weather forecasting information, and private keys are kept secret to be adopted by every other node inside the network.
- The role of RSU is to implement the routing strategy to obtain minimum hop count, less time, secure content delivery, and network throughput.

### 3.3. Routing Strategy

The primary goal of this work was to establish effective routing for secure weather forecasting information distribution. The chosen routing algorithm aimed to achieve high packet delivery rates while minimizing end-to-end delays. System analysis was conducted to address the network design and operational challenges. Two primary challenges for efficient routing were node connectivity and channel reliability.

In terms of the node connectivity, we considered two devices in the network to be active, and the other as a dumb type. Smart devices are referred to as such in the case of providing access to the data and onboard sensors of the vehicles, and others are transmitted toward less accessible devices. The ratio of message exchange in low mobility networks is very high compared to the risks associated with packet drops due to low network performance. Due to packet drops, the waiting time of the network performance expired due to network connectivity issues and performance enhancements.

Channel reliability is another factor in network routing. Whenever the network density is high, limited medium bandwidth is caused. Messaging is another issue which is faced due to the overwhelming of network performance parameters. Due to the high availability of the network parameters, the channel coverage and bandwidth performance enhancements are limited due to the limited packet rates. The channel topology is flexible due to the wireless activity and performance enhancements. The channel’s reliability is maximum with the maximum availability of network parameters. For instance, vehicles may not be able to receive GPS signals in certain areas, beacon messages may collide with other messages, and vehicles may be selfish and refuse to broadcast the message.

#### 3.3.1. Routing Protocol Strategy

The main factors to be considered for routing strategies among network communication are communication among nodes, hop count, weather data reliability, and distance between vehicles.

#### 3.3.2. Adopted Routing Method

We combined the time and location dependency of the vehicles in this routing technique. Whenever the bandwidth inside the network was limited, the number of packets transmitted was also limited due to limited bandwidth strategies. In this technique, we considered node mobility and network density to offer the efficient routing of weather forecasting information. We considered approximately 70 km/h, with two-direction lanes where, approximately, the vehicle travelled from 16 to 19 m in one second. The reliable distance and discovery of every node density from one vehicular distance towards another, and from one part towards others other, was calculated from distance inside the network. If the waiting time for the message during travelling expired, then the message would automatically drop.

Inside the network, communication among the vehicles was in both directions. The relay of communication described meant that the opposite-direction communication, with the reliable delivery of packets forwarding and packets delivery network evaluation, performed better. The senders were considered static vehicles with the same or opposite directions for sending the request or response packets. In this scenario, we adopted GPS-based connectivity and location settings, because GPS provides an accurate vehicle position in its reliable network services delivery ratio [30].

#### 3.3.3. Multi-Hop Routing Method

To achieve a high level of network connectivity with efficient weather data broadcasting, in terms of supersession and network connectivity, we selected the minimum number of intermediate nodes for effective network connectivity and reliability node values. The network coverage also decreased whenever the number of relay messages decreased inside the network. To increase the speed and reliability of the network connectivity, we set up five hops in each routing for network connectivity values and provided efficient network services. Each weather forecasting information packet had several relay nodes attached with a counter to select and count the number of intermediate nodes. Every intermediate node checked the hop counter threshold. If it became lower, the message was received or forwarded to the next hop for the destination node. The protocol worked with efficient routing information when this number of incident nodes occurred. The sources and destination nodes only shared the blockchain-based encrypted data using the public and private keys, and kept secure the number of relay packets it received with the highest accuracy values. Figure 3 shows the routing node. Intermediate nodes, from sender to recipient nodes, were selected based on their abilities to participate in the data processing, the number of tasks currently processing, communication, and, more specifically, the closest range in the sending device. When the sender node sent the device, it became the predecessor, and the next node became the sending node. Then, the new sending node searched for the next hop. The proposed weather forecasting data and information flow technique determined the minimum number of hop counts.

#### 3.3.4. Intermediate Node Transmission in Routing

**Figure 6:**
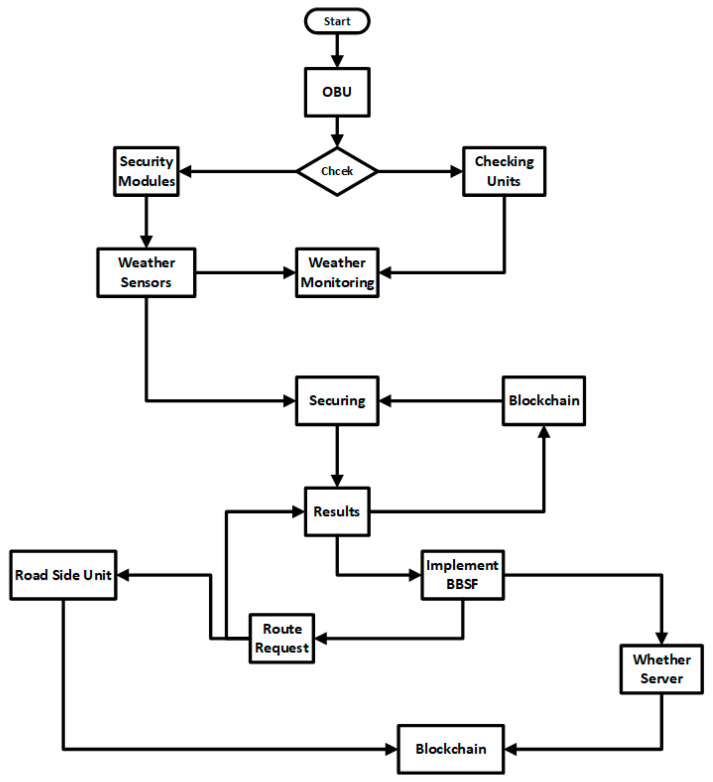
Logical Workflow of Proposed Methodology.

In routing, the intermediate node receives messages from its previous node and forwards them to the next vehicle when the node threshold is at its original position. The intermediate node calculates the distance from the distance and radius of its intermediate nodes when receiving the packets. A waiting time is calculated when a vehicle waits to receive the messages from its other intermediate node’s waiting time. A reservation ratio is maintained when the intermediate node receives duplicate messages from its other nodes with a limited number. The previous intermediate nodes only choose those nodes which do not receive the packet in its previous version of the message transmission range. This is called the emergency messages in our proposed routing protocol information. Figure 4, Figure 5 and Figure 6 shows the message received and forwarded using time and location based on the proposed routing approach.

Algorithm 1 presents packet forwarding using the proposed routing methodology for weather forecasting information. In this algorithm, the sender acts as a forwarding node that sends the broadcast messages to its corresponding nodes, and other nodes act as intermediate nodes that send packets to the next nodes. Table 1 uses the mostly used terminologies in the research work.

**Algorithm 1:** Routing with Packet Forwarding**Input:** Packets, packet_id (every packet has an ID), n (number of hop count), min_res (smallest waiting time), di, the distance of the node from the previous node, R (transmission range), c (duplication reception of messages). **Output:** Routing, Packet received and drop Steps:              $Node\;Mi\;receive\;a\;new\;packet\left(packet_{id},\;packet\right)$ Get GPS coordinates If (packet direction is from back)    $check\;hop\;count\;i.e.,\;\left(n=ni\right)$
    if (*n* ≤ 5)          waiting_time() ← compute()               if (initially receipt of packet i.e., packet_id = $id_{i}$)                      set (waiting time) ← lower_limit()               else                        increase (packet reservation ratio) ← packet_id       expire (waiting time, vehicle $m_{i}$)             if (waiting time(packet_id) ← min_reserved()                  Get_info (neighbour node, GPS)                 Packet_forward (location(previous forward, packet_id = $id_{i}$)                     If (*n* ≤ 4)                           Forward (vehicle-front, same direction)                     else (*n* = 5)                            forward (alternate-path)                else (packet_id = $id_{i}$ ≠ min-waiting-time)                     drop(packet_id = $id_{i}$)           else(*n* > 5)               drop(packet_id = $id_{i}$)      else            drop(packet_id = $id_{i}$) End

### 3.4. Weather Forecasting Information Security

Hyperledger Sawtooth is a permissioned blockchain framework for distribution and wireless application platforms such as VANETs. The purpose of using Hyperledger Sawtooth is to adopt the blockchain by simplifying the application process and separating the central layer from the application system processes. In this research, we adopted validator, REST API, transactions, and data as clients. Validator accepts the transaction requests. After receiving the requests, these are forwarded to the transaction processor for further action. The validator decides on a new block for every piece of information coming from the sensory modules of the systems. The validator keeps the state consistent from the number of items received for the validator. The transaction processes are used to separate the business logic from the application logic. The REST API works as a bridge between the vehicle clients and validators. Figure 6 shows the application and business logic development and sampling process.

### 3.5. Network Design and Operational Challenges

During the model implementation and analysis, the network design and its operational challenges were mentioned in the system implementation.

- **Bandwidth constraints:** The transmission of weather data in a vehicular ad hoc network (VANET) can cause bandwidth constraints, particularly during heavy traffic.
- **Security challenges:** Given that the weather data are sensitive, there is a high risk of data interception or theft. Implementing security measures will be crucial in guaranteeing that the data remains secure.
- **Scalability:** The implementation of blockchain technology in a VANET context can be challenging, particularly when it comes to scalability. The network may experience issues if there is an increase in the number of nodes or if the network traffic increases.
- **Reliability:** The reliability of the blockchain-based secure weather forecasting system will depend on the underlying routing protocol in VANETs. If the routing protocol is inefficient, this can result in packet loss or delay, impacting the system’s overall reliability.
- **Privacy concerns:** Blockchain technology can raise privacy concerns, particularly if the network is open to third-party nodes. The risk of exposing personal data and implementing privacy measures is crucial.
- **Maintenance:** Implementing the BBSF system will require regular maintenance to ensure the network is functioning correctly. This includes monitoring the system for any potential issues and ensuring that updates are applied promptly.
- **Interoperability:** The BBSF system must be interoperable with other VANET systems to ensure it can communicate with other vehicles on the road. This may require the use of common communication standards or protocols.

## 4. Discussion and Results

This section covers the outcomes of the proposed model in fulfilling the primary research objectives. The study examines the network routing and weather security parameters in VANETs, including hop count, latency, packet delivery ratio, and network overhead. These are the significant factors for accessing information.

### 4.1. Scenario

We tested our proposed model by implementing it in NS-2, using an environment with an HP Foliobook Pro with a 10th generation core i5 CPU, 8 GB of RAM, 1 TB HDD, Linux OS, and the NS-2 setup [31]. Table 1 describes the environment setup and variables. Our VANET network was designed to ensure that the proposed VANET devices behaved effectively. We selected four parameters to study the performance of the proposed system, and we discussed the proposed algorithms and methodologies in a previous version of our work. To evaluate the results, we compared them with the current AODV and CBDRP techniques. The sender and receiver were in a concurrent format with effective monitoring and provided a reliable service delivery for packet sending and receiving. Intermediate nodes were deployed to forward the packets and receive the packets from sender to receiver nodes. This section of the thesis focused on the execution of the routing algorithm based on the proposed parameters. The packets were forwarded from sender to receiver through intermediate nodes. Table 2 shows the parameters with the values used in the simulation process [32,33].

### 4.2. End-to-End Latency

**Figure 7:**
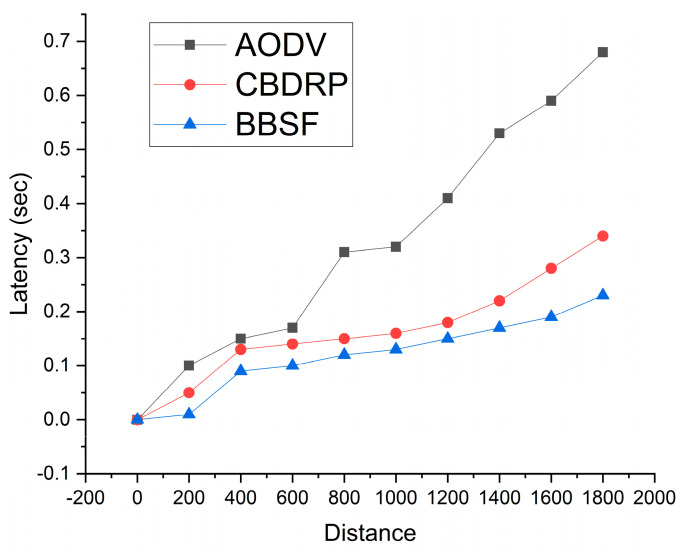
Latency of the network with distance.

End-to-end latency in the network is the actual time when packets are transmitted inside the network. In this work situation, we took the latency as one, ensuring the packet forwarding and sending delay inside the network. In Figure 7, the distance of AODV shows higher hop counts than the previous section, so the latency is also greater than 7. This shows the complete ratio, explaining that CBDRP shows smaller latency than AODV. At a longer distance, the AODV and CBDRP are prone to establishing links among the nodes. On the other hand, the proposed BBSF approach shows the lowest latency of these techniques, which shows that the proposed approach effectively provides the simulation results for latency values as shown in Figure 7.

### 4.3. Network Overhead

**Figure 8:**
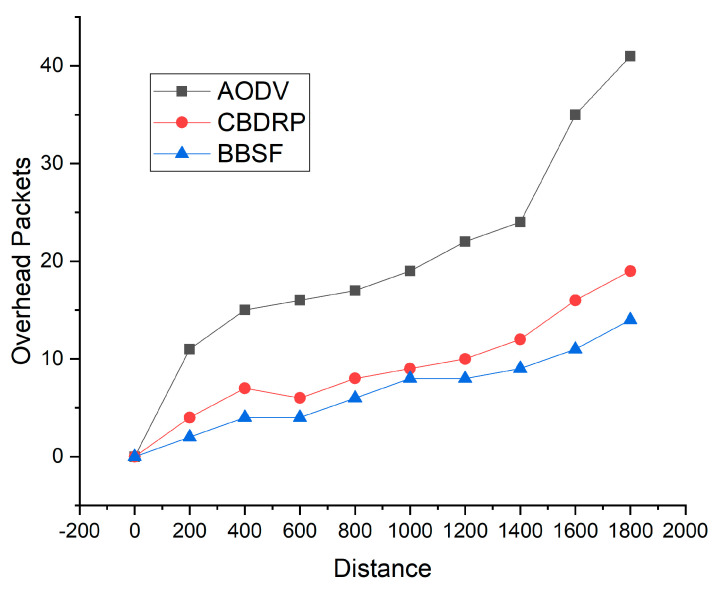
Routing overhead with distance among vehicles.

Figure 8 shows the routing overhead by comparing BBSF to AODV and CBDRP techniques. In this approach, the overhead is involved in sending and receiving packets for the packet transfer rates inside the network. We compared the number of overhead packets with the distance for routing in the proposed technique. The distance was taken among vehicles used as intermediate nodes in the network. In the proposed BBSF approach, the overhead packets increased slowly with the increasing distance. This shows that the packet overhead according to distance is better in the proposed approach than the AODV and CBDRP approach. With distance increase, the overhead in packet delivery increased in AODV and CBRP techniques.

### 4.4. Packets Delivery Ratio

**Figure 9:**
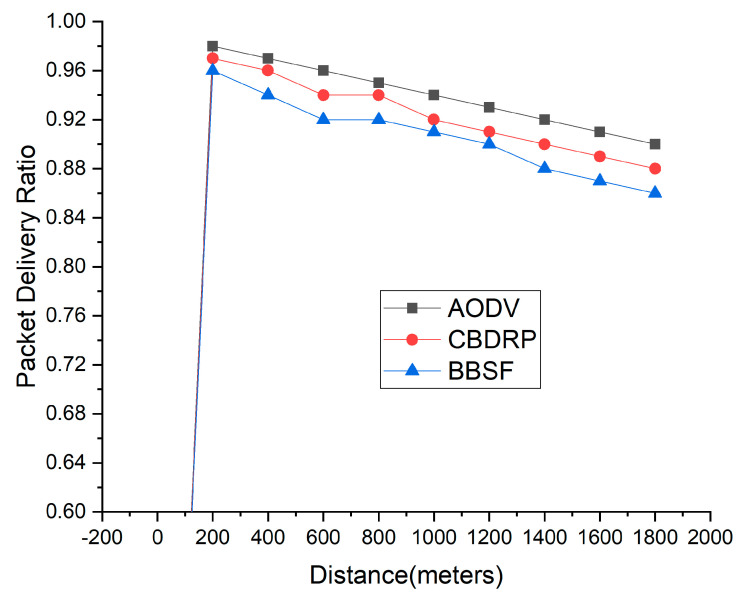
Packet Delivery Ratio of Proposed Technique with Distance.

The packet delivery ratio is the percentage of packets received over packets sent to the sending nodes. The proposed approach’s delivery ratio is measured according to the distance inside the network. Figure 9 shows the packet delivery ratio results from the proposed technique. With the distance increase among the vehicles, the AODV and CBDRP performance is slightly lower and provides lesser communication overhead. On longer distances, the overhead time and ratio are also lessened, which provides a shorter overhead time compared to packet delivery. The performance goes down when delivery ration goes down but, inside the proposed architecture, the performance was lower compared to the existing techniques. The BBSF performs better in comparison to the existing technique.

### 4.5. Hop Count in the Network

**Figure 10:**
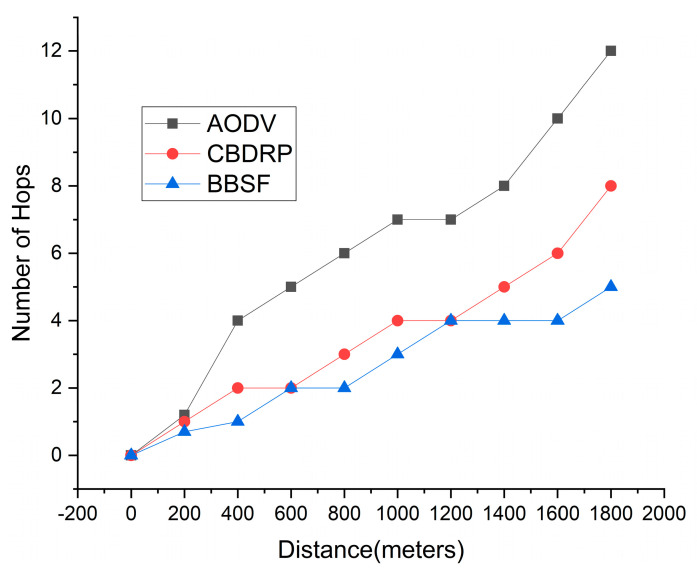
Network Hop Count.

Hop count means the intermediate nodes followed to reach the destination or source vehicle. The average number of hops counts is the packets that reach their destination from time to time, over the network performance. In Figure 10, the BBSF shows lower hop counts, as mentioned in the proposed methodology. We only considered five hop counts for the packets sent from source to destination. Other techniques, such as AODV and CBDRP, show fewer hop counts with the minimum number of packets sent and received from the source to the destination folder, a broad concept for the provision of hop count and the number of packets sent from source to destination nodes.

## 5. Conclusions, Limitations, and Future Directions

Our research concluded that weather forecasting information is necessary for safety and driving efficiency. The secure data distribution among vehicles inside the VANET environment ensures the reliable delivery of the network services and packet delivery. Moreover, routing such information is very important, because a lower hop count and less efficient routing make better weather prediction and are useful for human lives. We present a novel blockchain-based secure weather forecasting and routing framework that efficiently routes information securely through blockchain technology. The routing algorithm efficiently routes the requested data from source to destination with minimum hop counts. We simulate the proposed framework using a blockchain-based environment in NS-2 based on the hop count, network latency, packet delivery ratio, and network overhead. Routing the correct weather forecasting information towards the correct destination is a challenging task achieved through routing in the proposed architecture. We set up a weather forecasting server which stores the information. We added the blockchain-based Hyperledger Sawtooth framework to provide security for our weather data. After adopting the security, the public key is distributed to the network and the private key is only exchanged with the destination node. Finally, we adopt the routing through an efficient routing algorithm to adopt and provide effective and logical routing schemes. The results are taken through an OMNeT++ simulator with the crypto secure library to secure the content. This outperformed the evaluations for hop count, latency, packet delivery ratio, and network overhead. The practical results show that the proposed BBSF technique efficiently contributed to the routing field when routing the secure routing in the VANET environment. The BBSF technique showed fewer hop counts, a lower network latency, efficient packet delivery ratio, and a shorter network overhead time for packets distributed to the correct destination vehicle.

The challenges/limitations of the proposed model/method are illustrated below:

- The proposed method is only limited to weather forecasting information routing.
- The routing is only based on near-vehicle concepts, and we did not apply any machine learning technique to the route-request response mechanism.
- The challenge of implementing 5G technology was faced during the simulation work of the proposed model, but we successfully implemented the proposed system.

In the future, a machine learning-based routing technique can be adopted to improve the minimization of hop count, network latency, and throughput values. The machine learning-based framework effectively evolved the performance of the system. Moreover, secure weather forecasting information can be adopted in railway warning systems using the Wi-Fi transmission sensor network.

## Figures and Tables

**Table 1:** Notations with descriptions used in algorithms and equations.

S. No.	Notation	Description
1	Packets	The weather forwarding data from the source to the destination vehicle
2	packet_id	Every individual weather forecasting data packet has a simplified id which is unique for every individual packet in the network.
3	n (hop count)	Number of hop counts from source to destination nodes.
4	min_res	The minimum reserved waiting time is almost the smallest and provides a unique value to the provided packets.
5	di	Distance of intermediate vehicles from source to destination (*n* = 1, 2, 3, ……, m)
6	R	Total transmission range from source to destination node.
7	C	Duplication of the messages originated from the source. The duplication is based on the point of view of different aspects.

**Table 2:** Parameters and their values for the proposed methodology.

S. No.	Parameter	Value	
1.	Simulation time	1000 s	
2.	Simulation area size	Region 1	Region 2
		1000 ×1000 m	2500 × 2500 m
3.	Number of vehicles	0 in Region 1 100 in Region 2	
4.	Vehicle time for a drive	150 s	
5.	Wireless area (Single RSU)	200 m	
6.	Cache values	0 s to 200 s	
7.	Radio	802.11b	
8.	Road condition	New entry (One-way road)	
9.	RSUs	10	
10.	RSU broadcast time interval	50 s	
11.	Breakdowns duration for vehicles	120 s	
12.	The number of simulations run	150	

## Data Availability

No copyright permission is required in the figures and tables in this manuscript.

## References

[1] Al-Heety O.S., Zakaria Z., Ismail M., Shakir M.M., Alani S., Alsariera H. (2020). A comprehensive survey: Benefits; services; recent works; challenges; security, and use cases for sdn-vanet. IEEE Access.

[2] Singh G.D., Prateek M., Kumar S., Verma M., Singh D., Lee H.N. (2022). Hybrid genetic firefly algorithm-based routing protocol for VANETs. IEEE Access.

[3] Hussain R., Hussain F., Zeadally S. (2019). Integration of VANET and 5G Security: A review of design and implementation issues. Future Gener. Comput. Syst..

[4] Ali Q.I. (2018). GVANET project: An efficient deployment of a self-powered, reliable and secured VANET infrastructure. IET Wirel. Sens. Syst..

[5] Xiao L., Ding Y., Jiang D., Huang J., Wang D., Li J., Poor H.V. (2020). A reinforcement learning and blockchain-based trust mechanism for edge networks. IEEE Trans. Commun..

[6] Goudarzi F., Asgari H., Al-Raweshidy H.S. (2018). Traffic-aware VANET routing for city environments—A protocol based on ant colony optimization. IEEE Syst. J..

[7] Soni M., Rajput B.S., Patel T., Parmar N. (2021). Lightweight vehicle-to-infrastructure message verification method for VANET. Data Science and Intelligent Applications.

[8] Waheed A., Shah M.A., Khan A., Jeon G. (2021). An infrastructure-assisted job scheduling and task coordination in volunteer computing-based VANET. Complex Intell. Syst..

[9] Azzoug Y., Boukra A. (2021). Bio-inspired VANET routing optimization: An overview. Artif. Intell. Rev..

[10] Ali I., Hassan A., Li F. (2019). Authentication and privacy schemes for vehicular ad hoc networks (VANETs): A survey. Veh. Commun..

[11] Kudva S., Badsha S., Sengupta S., La H., Khalil I., Atiquzzaman M. (2021). A scalable blockchain based trust management in VANET routing protocol. J. Parallel Distrib. Comput..

[12] Ghori M.R., Sadiq A.S., Ghani A. (2018). VANET routing protocols: Review; implementation; analysis. J. Phys. Conf. Ser..

[13] Phull N., Singh P., Shabaz M., Sammy F.J.S., Networks C. (2022). Enhancing vehicular ad hoc networks’ dynamic behavior by integrating game theory and machine learning techniques for reliable and stable routing. Secur. Commun. Netw..

[14] Ahamed A., Vakilzadian H. (2018). Issues and challenges in VANET routing protocols. Proceedings of the 2018 IEEE International Conference on Electro/Information Technology (EIT).

[15] Singh G.D., Kumar A., Dumka A. (2022). Routing Challenges in Vehicular Ad-hoc Network and Significance of Swarm Intelligence for Efficient Routing. Advances in Information Communication Technology and Computing: Proceedings of AICTC 2021.

[16] Taleb A.A. (2018). VANET Routing Protocols and Architectures: An Overview. J. Comput. Sci..

[17] Marwah G.P.K., Jain A., Malik P.K., Singh M., Tanwar S., Safirescu C.O., Mihaltan T.C., Sharma R., Alkhayyat A. (2022). An Improved Machine Learning Model with Hybrid Technique in VANET for Robust Communication. Mathematics.

[18] Jiang H., Guo H., Chen L. (2008). Reliable and efficient alarm message routing in VANET. Proceedings of the 2008 the 28th International Conference on Distributed Computing Systems Workshops.

[19] Dave M., Rastogi V., Miglani M., Saharan P., Goyal N. (2022). Smart fog-based video surveillance with privacy preservation based on blockchain. Wirel. Pers. Commun..

[20] Sharma S., Kumar A., Bhushan M., Goyal N., Iyer S.S. (2021). Is blockchain technology secure to work on?. Blockchain and AI Technology in the Industrial Internet of Things.

[21] Rana A., Sharma S., Nisar K., Ibrahim A.A.A., Dhawan S., Chowdhry B., Hussain S., Goyal N. (2022). The Rise of Blockchain Internet of Things (BIoT): Secured, Device-to-Device Architecture and Simulation Scenarios. Appl. Sci..

[22] Chauhan H., Gupta D., Gupta S., Singh A., Aljahdali H.M., Goyal N., Noya I.D., Kadry S. (2021). Blockchain enabled transparent and anti-counterfeiting supply of COVID-19 vaccine vials. Vaccines.

[23] ul Hassan M., Al-Awady A.A., Ali A., Iqbal M.M., Akram M., Khan J., AbuOdeh A.A. (2023). An efficient dynamic decision-based task optimization and scheduling approach for microservice-based cost management in mobile cloud computing applications. Pervasive Mob. Comput..

[24] Sohail R., Saeed Y., Ali A., Alkanhel R., Jamil H., Muthanna A., Akbar H. (2023). A Machine Learning-Based Intelligent Vehicular System (IVS) for Driver’s Diabetes Monitoring in Vehicular Ad-Hoc Networks (VANETs). Appl. Sci..

[25] Rashid K., Saeed Y., Ali A., Jamil F., Alkanhel R., Muthanna A. (2023). An Adaptive Real-Time Malicious Node Detection Framework Using Machine Learning in Vehicular Ad-Hoc Networks (VANETs). Sensors.

[26] Nazar K., Saeed Y., Ali A., Algarni A.D., Soliman N.F., Ateya A.A., Muthanna M.S.A., Jamil F. (2022). Towards Intelligent Zone-Based Content Pre-Caching Approach in VANET for Congestion Control. Sensors.

[27] Jamil F., Jamil H., Ali A. (2022). Spoofing Attack Mitigation in Address Resolution Protocol (ARP) and DDoS in Software-Defined Networking. J. Inf. Secur. Cybercrimes Res. (JISCR).

[28] Ali A., Iqbal M.M., Jabbar S., Asghar M.N., Raza U., Al-Turjman F. (2022). VABLOCK: A blockchain-based secure communication in V2V network using icn network support technology. Microprocess. Microsyst..

[29] Ahmad S., Khan S., Jamil F., Qayyum F., Ali A., Kim D. (2022). Design of a general complex problem-solving architecture based on task management and predictive optimization. Int. J. Distrib. Sens. Netw..

[30] Shah P., Kasbe T. (2021). A review on specification evaluation of broadcasting routing protocols in VANET. Comput. Sci. Rev..

[31] Tseng Y.-T., Jan R.-H., Chen C., Wang C.-F., Li H.-H. (2010). A vehicle-density-based forwarding scheme for emergency message broadcasts in VANETs. Proceedings of the 7th IEEE International Conference on Mobile Ad-hoc and Sensor Systems (IEEE MASS 2010).

[32] Lyu F., Zhu H., Zhou H., Xu W., Zhang N., Li M., Shen X. (2017). SS-MAC: A novel time slot-sharing MAC for safety messages broadcasting in VANETs. IEEE Trans. Veh. Technol..

[33] Tonguz O., Wisitpongphan N., Bai F., Mudalige P., Sadekar V., Vanet B.I. (2007). 2007 Mobile Networking for Vehicular Environments.

